# Supplementary material for: A Multigraph-Based Representation of Hi-C Data
Source: Genes (Basel). 2022 Nov 23;13(12):2189. doi: 10.3390/genes13122189 (PMC9778156; doi:10.3390/genes13122189)
Supplement: Supplementary file 1 [file genes-13-02189-s001.zip › Supplementary Table 1.pdf]

Supplementary Table S1: The result of the GO enrichment analysis. Genes and GO terms are shown by clusters as per Error! Reference source not found.. **Columns with grey background shows GO terms of the most relevance as per Revigo.**

Cluster	Genes	GO	p-value	Revigo	GO name
1	HORVU.MOREX.r2.2HG0084010	GO:0016829	0.000326587	GO:0007142	male meiosis II
	HORVU.MOREX.r2.2HG0084040	GO:0010333	0.022393533	GO:0006777	Mo-molybdopterin cofactor biosynthetic process
	HORVU.MOREX.r2.2HG0084050	GO:0048544	0.042311555	GO:0009767	photosynthetic electron transport chain
	HORVU.MOREX.r2.7HG0528980	GO:0007142	0.002447013	GO:0048544	recognition of pollen
	HORVU.MOREX.r2.7HG0611230	GO:0030170	0.03930111		
	HORVU.MOREX.r2.7HG0611240	GO:0006777	0.002853725	GO:0042651	thylakoid membrane
	HORVU.MOREX.r2.7HG0611250	GO:0030151	0.00203998		
	HORVU.MOREX.r2.7HG0611260	GO:0102867	0.000408639		
	HORVU.MOREX.r2.7HG0611270	GO:0008265	0.000408639		
		GO:0009767	0.011320919		
		GO:0042651	0.002853725		
		GO:0045156	0.004882481		
6	HORVU.MOREX.r2.3HG0251230	GO:0003677	0.01783533	GO:0006351	transcription, DNA-templated
	HORVU.MOREX.r2.4HG0323870	GO:0006351	0.017564942	-	
	HORVU.MOREX.r2.5HG0382510	GO:0003899	0.009321586	-	
	HORVU.MOREX.r2.5HG0438360	GO:0005347	0.003936803	-	
5	HORVU.MOREX.r2.1HG0009390	GO:0046983	0.010749531	GO:0006397	mRNA processing
	HORVU.MOREX.r2.1HG0009370	GO:0008171	2.01E-07	GO:0009630	gravitropism
	HORVU.MOREX.r2.1HG0009380	GO:0003735	0.049316596	GO:2000012	regulation of auxin polar transport
	HORVU.MOREX.r2.2HG0119720	GO:0006412	0.041744365	GO:0009772	photosynthetic electron transport in photosystem II
	HORVU.MOREX.r2.2HG0119730	GO:0019843	0.029905686	GO:0006412	translation
	HORVU.MOREX.r2.4HG0328900	GO:0005524	0.013941887	GO:0019684	photosynthesis, light reaction
	HORVU.MOREX.r2.4HG0348720	GO:0009507	4.85E-10		
	HORVU.MOREX.r2.5HG0351700	GO:0006397	1.22906E-05	GO:0009539	photosystem II reaction center
	HORVU.MOREX.r2.5HG0351720	GO:0045156	0.009899508	GO:0009507	chloroplast
	HORVU.MOREX.r2.5HG0351740	GO:0019684	0.017197097	GO:0015935	small ribosomal subunit
	HORVU.MOREX.r2.5HG0351750	GO:0009772	0.007442868	GO:0016592	mediator complex
	HORVU.MOREX.r2.5HG0351810	GO:0009523	0.046080582		
	HORVU.MOREX.r2.5HG0447040	GO:0009539	0.006621295		
	HORVU.MOREX.r2.6HG0458780	GO:0015935	0.035360437		
	HORVU.MOREX.r2.6HG0525460	GO:0016592	0.039217903		
	HORVU.MOREX.r2.6HG0525530	GO:0003712	0.040751891		
	HORVU.MOREX.r2.7HG0531040	GO:0009630	0.003321493		
	HORVU.MOREX.r2.UnG0626200	GO:2000012	0.000832413		
	HORVU.MOREX.r2.UnG0631430				
	HORVU.MOREX.r2.UnG0634820				
	HORVU.MOREX.r2.UnG0635060				
	HORVU.MOREX.r2.UnG0636130				
3	HORVU.MOREX.r2.1HG0000220	GO:0030246	1.31E-07	GO:0006952	defense response
	HORVU.MOREX.r2.1HG0000210	GO:0006952	0.035307495	GO:0016192	vesicle-mediated transport
	HORVU.MOREX.r2.2HG0088720	GO:0048046	2.41E-08	GO:0006886	intracellular protein transport
	HORVU.MOREX.r2.3HG0224110	GO:0006886	0.040797895		
	HORVU.MOREX.r2.7HG0534690	GO:0016192	0.026098537	GO:0048046	apoplast

	HORVU.MOREX.r2.7HG0534710	GO:0030117	0.005886245	GO:0030117	membrane coat
	HORVU.MOREX.r2.7HG0541120				
	HORVU.MOREX.r2.UnG0627550				
	HORVU.MOREX.r2.UnG0629750				
	HORVU.MOREX.r2.UnG0633140				
	HORVU.MOREX.r2.UnG0634230				
4	-				
2	HORVU.MOREX.r2.2HG0081910	GO:0016829	0.000160326	GO:0000724	double-strand break repair via homologous recombination
	HORVU.MOREX.r2.2HG0082000	GO:0010333	0.000118978	GO:0009908	flower development
	HORVU.MOREX.r2.3HG0255950	GO:0000287	0.041151279	GO:0030154	cell differentiation
	HORVU.MOREX.r2.4HG0330120	GO:0048544	0.030163501	GO:0048544	recognition of pollen
	HORVU.MOREX.r2.5HG0445110	GO:0009908	0.002296102		
	HORVU.MOREX.r2.5HG0445120	GO:0030154	0.007425815	GO:0031519	PcG protein complex
	HORVU.MOREX.r2.7HG0544320	GO:0000724	0.005721514		
		GO:0018024	0.010253908		
		GO:0031519	0.000862212		
